# The psychometric properties of the Quality of Life in Neurological Disorders (Neuro-QoL) measurement system in neurorehabilitation populations: a systematic review

**DOI:** 10.1186/s41687-024-00743-7

**Published:** 2024-09-18

**Authors:** Rebecca Ataman, Rehab Alhasani, Line Auneau-Enjalbert, Adria Quigley, Henry Ukachukwu Michael, Sara Ahmed

**Affiliations:** 1 School of Physical and Occupational Therapy, McGill University, Montréal, Québec Canada; 2 Centre for Interdisciplinary Research in Rehabilitation of Greater Montréal, Montréal, Québec Canada; 3 Clinical Institutes and Quality Programs, Ontario Health, Toronto, Ontario Canada; 4 Department of Rehabilitation Sciences, College of Health and Rehabilitation Sciences, Princess Nourah bint Abdulrahman University, Riyadh, Saudi Arabia; 5 Constance Lethbridge Rehabilitation Center, CIUSSS Centre-Ouest de l’Île de Montreal, Montreal, Quebec Canada; 6 School of Physiotherapy, Dalhousie University, Forrest Building, 5869 University Avenue, PO Box 15000, Halifax, Nova Scotia B3H 4R2 Canada; 7 Nova Scotia Health Authority, Nova Scotia Rehabilitation and Arthritis Centre, 1341 Summer St, Halifax, Nova Scotia B3H 4K4 Canada; 8 Division of Experimental Medicine, McGill University, Montreal, Quebec Canada; 9 Center for Outcomes Research and Evaluation (CORE), Research Institute of the McGill University Health Center, Montreal, Quebec Canada; 10 Clinical Epidemiology, Center for Outcome Research and Evaluation (CORE), McGill University Health Center Research Institute, Montreal, Quebec Canada

**Keywords:** Patient reported outcome measures, Nervous system diseases, Psychometrics

## Abstract

**Objective:**

To systematically review the literature of existing evidence on the measurement properties of the Quality of Life in Neurological Disorders (Neuro-QoL) measurement system among neurorehabilitation populations.

**Data sources:**

The Consensus-based Standards for the selection of health Measurement Instruments (COSMIN) guided this systematic review in which we searched nine electronic databases and registries, and hand-searched reference lists of included articles.

**Study selection:**

Two independent reviewers screened selected articles and extracted data from 28 included studies.

**Data extraction:**

COSMIN’s approach guided extraction and synthesizing measurement properties evidence (insufficient, sufficient), and the modified GRADE approach guided synthesizing evidence quality (very-low, low, moderate, high) by diagnosis.

**Data synthesis:**

Neuro-QoL has sufficient measurement properties when used by individuals with Huntington’s disease, Multiple Sclerosis, Parkinson’s disease, stroke, lupus, cognitive decline, and amyotrophic lateral sclerosis. The strongest evidence is for the first four conditions, where test-retest reliability, construct validity, and responsiveness are nearly always sufficient (GRADE: moderate-high). Structural validity is assessed only in multiple sclerosis and stroke but is often insufficient (GRADE: moderate-high). Criterion validity is sufficient in some stroke and Huntington’s disease domains (GRADE: high). Item response theory analyses were reported for some stroke domains only. There is limited, mixed evidence for responsiveness and measurement error (GRADE: moderate-high), and no cross-cultural validity evidence

**Conclusions:**

Neuro-QoL domains can describe and evaluate patients with Huntington’s disease, multiple sclerosis, Parkinson’s disease, and stroke, but predictive validity evidence would be beneficial. In the other conditions captured in this review, a limited number of Neuro-QoL domains have evidence for descriptive use only. For these conditions, further evidence of structural validity, measurement error, cross-cultural validity and predictive validity would enhance the use and interpretation of Neuro-QoL.

**Supplementary Information:**

The online version contains supplementary material available at 10.1186/s41687-024-00743-7.

## Introduction

The need for adult rehabilitation is expected to increase as people live longer, and as the prevalence of chronic disease and disability rises [1]. In 2019, about 255 million people globally required neurological rehabilitation, making individuals with neurological conditions one of the largest patient groups requiring rehabilitation services. Furthermore, individuals with neurological conditions have one of the highest ‘disability weights’ or calculated disease severity [2]. Therefore, it is imperative that these individuals receive the best possible rehabilitation care. Targeted and robust patient-reported outcome measures can positively impact patient care by improving patient-provider communication and offering standardized assessments to identify areas to target in rehabilitation and monitor outcomes [3, 4].

The Quality of Life in Neurological Disorders (Neuro-QoL) measurement system is a patient-reported measurement system that assesses health-related quality of life in populations with neurological conditions, especially stroke, multiple sclerosis, amyotrophic lateral sclerosis, Parkinson’s disease, epilepsy, and muscular dystrophy [5, 6]. Neuro-QoL can be used to optimize patient care by obtaining a patient’s experience of their symptoms, treatment side effects, functioning, and well-being. In 2011, Neuro-QoL-version 1.0 was first published, encompassing 17 domains within physical, mental, and social health [5, 6]. Version 2.0 followed in 2015, with changes to some domains and statistics [7]. In each version, domains are structured as either scales or item banks, allowing for efficient measurement through fixed-length short forms and/or computerized adaptive testing (CATs) [8].

Since its initial publication over a decade ago, there has been considerable uptake of Neuro-QoL by researchers and clinicians. There have been at least 200 publications in which authors used Neuro-QoL [9], including those recommending the use of Neuro-QoL as part of a core outcome set [10], standardized survey [11] or within clinical trials [12]. Despite the increasing use of Neuro-QoL, there is no synthesis of its available psychometric evidence to inform evidence-based use in adults receiving rehabilitation care. Existing syntheses of Neuro-QoL are limited, focusing only on specific aspects like a single domain such as fatigue [13], or a particular diagnosis such as traumatic brain injury [14] or Parkinson’s disease [15]. There is a need for a comprehensive synthesis of the current evidence of Neuro-QoL within all neurological rehabilitation populations to highlight psychometric strengths, weaknesses, and gaps, and inform the use of Neuro-QoL in clinical practice.

Thus, the objective of this review was to systematically review the literature of existing evidence on the measurement properties of Neuro-QoL measures among neurorehabilitation populations.

## Methods

This study was part of a larger systematic review encompassing Neuro-QoL and related measurement systems: Patient Reported Outcomes Measurement Information System (PROMIS), Traumatic Brain Injury Quality of Life (TBI-QoL) and Spinal Cord Injury Quality of Life (SCI-QoL). While we originally intended to report our findings from all these measurement systems in one consolidated manuscript, the volume of information precluded this option. Thus, here we focus on the measurement properties of the Neuro-QoL according to the COnsensus-based Standards for the selection of health Measurement Instruments (COSMIN) 2018 guidelines [16]. COSMIN has not developed comprehensive guidelines for extracting and rating of Rasch analyses. Therefore, the research team applied criteria previously developed and used in an earlier systematic review to inform Rasch synthesis and interpretation [17]. We report this manuscript in accordance with the PRISMA guidelines for systematic reviews [18].

### Literature search and eligibility

Two reviewers independently searched electronic databases (MEDLINE, EMBASE, PsycINFO and HaPI (Ovid), CINAHL (EBSCO), Cochrane Library and Web of Science), and clinical trials registries (ISRCTN Registry and ClinicalTrials.gov) from inception to March 23^rd^ 2024. The search strategy (Psychometric properties AND (Neuro-QoL OR PROMIS OR TBI-QoL OR SCI-QoL) AND Rehabilitation Conditions; MEDLINE search strategy in Appendix A) was developed using a measurement properties search filter validated by COSMIN [19] and drawing from other search strategies for COSMIN reviews by the same research team (e.g., [17])

After deduplication in EndNote X9 [20], two reviewers independently screened titles and abstracts, followed by the full texts of the manuscripts. We included peer-reviewed articles in English or French providing original data on Neuro-QoL, PROMIS, TBI-QoL, or SCI-QoL measurement properties, feasibility, or interpretability among any rehabilitation population—we did not impose restrictions on diagnosis (Table 1). We excluded articles that: (1) did not investigate the measurement properties of these measurement systems (e.g., used as an outcome measure only); (2) used these measurement systems to validate another measure [16]; (3) were published before 2004 (this being the year of the first PROMIS publication); (4) were posters or abstracts or (5) pediatric or (6) non-rehabilitation populations (e.g., mental health, focus on surgical modality such as for orthopedic injuries, etc). We resolved disagreements between reviewers by consensus or with another research team member when necessary. We hand-searched reference lists of all included articles for possible inclusion.

**Table 1:** COSMIN guidelines and extended Rasch criteria for evaluated measurement properties

	Measurement property	Definition	Data management and interpretation^1^
*COSMIN definitions of psychometric properties*	*Content validity*	The degree to which the content of a measure is an adequate reflection of the construct to be measured	*COSMIN synthesis:* Adequate if the development paper reported clear descriptions of the measurement aim, target population, dimensions measured, and item selection process Measure should be comprehensive, comprehensible and relevant according to clinicians/researchers, caregivers and patients
	*Structural validity*	The degree to which the scores of a measure are an adequate reflection of the dimensionality of the construct to be measured	*COSMIN synthesis:* Exploratory or confirmatory factor analysis with adequate model fit (e.g., Kaiser-Meyer-Olkin test 0.8–1.0, Bartlett’s test significant)
	*Internal consistency*	The degree of interrelatedness among the items	*COSMIN synthesis:* Cronbach’s alpha(s) ≥ 0.70 for each unidimensional scale or subscale AND at least low evidence for sufficient structural validity *Meta-analysis:* Weighted mean and range of results calculated for Cronbach’s alpha where possible
	*Cross-cultural validity*	The degree to which the performance of the items on a translated or culturally adapted measure are an adequate reflection of the performance of the items of the original version	*COSMIN synthesis:* No important differences found between group factors (such as age, gender, language) in multiple group factor analysis OR no important differential item functioning for group factors (McFadden’s R2 < 0.02)
	*Reliability*	The proportion of the total variance in the measurements which is due to ‘true’ differences between patients	*COSMIN synthesis:* Intra-class correlation coefficient or spearman’s correlation ≥ 0.70 OR Rater reliability: > 0.8 AND Rater separation: < 0.2 *Meta-analysis:* Weighted mean and range of results calculated for ICC where possible
	*Measurement error*	The systematic and random error of a patient’s score that is not attributed to true changes in the construct to be measured	*COSMIN synthesis:* Smallest detectable change < minimal important change
	*Criterion validity*	The degree to which the scores of a measure are an adequate reflection of a ‘gold standard’	*COSMIN synthesis:* For predictive validity, prediction should be clinically meaningful
	*Construct validity*	The degree to which the scores of a measure are consistent with hypotheses based on the assumption that the measure validly assesses the construct to be measured	*COSMIN synthesis:* The result is in accordance with the hypothesis 1. Correlations with (changes in) instruments measuring similar constructs should be ≥ 0.50. 2. Correlations with (changes in) instruments measuring related, but dissimilar constructs should be lower, i.e., 0.30–0.50. 3. Correlations with (changes in) instruments measuring unrelated constructs should be < 0.30. 4. Correlations defined under 1, 2, and 3 should differ by a minimum of 0.10. *Meta-analysis:* Weighted mean and range of results calculated for correlations to a measure.
	*Responsiveness*	The degree to which a measure can detect change over time in the construct to be measured	*COSMIN synthesis:* The result is in accordance with the hypothesis OR area under the curve ≥ 0.70. 1. Meaningful changes between relevant (sub)groups (e.g., patients with expected high vs low levels of the construct of interest)
*Rasch analysis*	*Structural validity*	*Defined by COSMIN as above*	*COSMIN synthesis:* Unidimensional, locally independent, monotonic with adequate model fit
	*Person and item reliability and separation*	The reproducibility of the person or item in a relative location on the measure (reliability) The spread of high and low performers or easy and hard items within a measure (separation)	*Narrative synthesis* Person or item separation > 2.0^2^ Person or item reliability > 0.8^2^
*IRT*	*Structural validity*	*Defined by COSMIN as above*	*COSMIN synthesis:* Unidimensional, locally independent, monotonic with adequate model fit

*COSMIN* COnsensus-based standards for the selection of health measurement instruments, *CTT* classical test theory, *SD* standard deviation, *ICC* intra-class correlation, *TCC* test characteristic curve

^1^See COSMIN manual and a priori hypotheses (Appendix B) for full details

^2^Boone WJ, Noltemeyer A. Rasch analysis: A primer for school psychology researchers and practitioners Cogent Educ. 2017;4(1):1416898

### Data extraction

After training sessions and 2–3 article pilots, two reviewers independently extracted data concerning the methods and results of the estimated measurement properties, study characteristics, and study population using the structured forms from COSMIN [16]. We consulted a third reviewer in the case of disagreement.

### Data analysis

Two reviewers independently assessed the measurement properties in each study. They rated content validity against COSMIN criteria [16, 21]. All other measurement properties were rated using Terwee and colleagues’ standards [22] as “sufficient” (+), “insufficient” (–), or “indeterminate” (?). When the COSMIN criteria for good measurement properties did not include the statistical test being used in the included study, we summarized and reported the measurement properties narratively.

The research team decided a priori that there is no gold standard measure that could be used to assess the criterion validity of Neuro-QoL. We set a priori hypotheses based on recommendations by de Vet and colleagues [23] for testing construct validity and responsiveness (Appendix B).

### Data synthesis

Based on their clinical experience, and the study and patient characteristics, the research team grouped the studies by measurement system (Neuro-QoL, PROMIS, TBI-QoL, SCI-QoL), domain (e.g., fatigue, stigma, etc,) and diagnosis (i.e., amyotrophic lateral sclerosis, cognitive decline or mild cognitive impairment, Huntington’s disease, lupus, stroke, multiple sclerosis, Parkinson’s disease, and mixed neurological conditions). In this manuscript, we report the synthesis of Neuro-QoL’s measurement properties.

Two reviewers independently summarized the results for each measurement property across studies (i.e., +/–/±/?). We gave an overall “sufficient” (+) or “insufficient” (–) rating if > 75% of measurement property results across studies were concurrent. We have an “inconsistent” (±) rating if no rating exceeded 75% and no appropriate explanation for inconsistency could be given. We gave an “indeterminate” (?) rating if the results did not achieve a sufficient or insufficient score (i.e., greater than 25% but less than 75% sufficient ratings).

### Quality assessment

Two independent reviewers assessed the methodological quality of individual studies using the COSMIN risk of bias checklist [16, 24]. Each checklist item is rated as “very good”, “adequate”, “doubtful” or “inadequate”. The overall rating of the methodological quality for a measurement property was based on the worst item rating [16, 24].

Two independent reviewers then graded the quality of evidence for each property per subgroup using the COSMIN modified Grading of Recommendations Assessment, Development and Evaluation (GRADE) approach [16, 25]. The quality of evidence was rated “high”, “moderate”, “low”, or “very low” after considering risk of bias, inconsistency, imprecision, and indirectness.

## Results

**Fig. 1:**
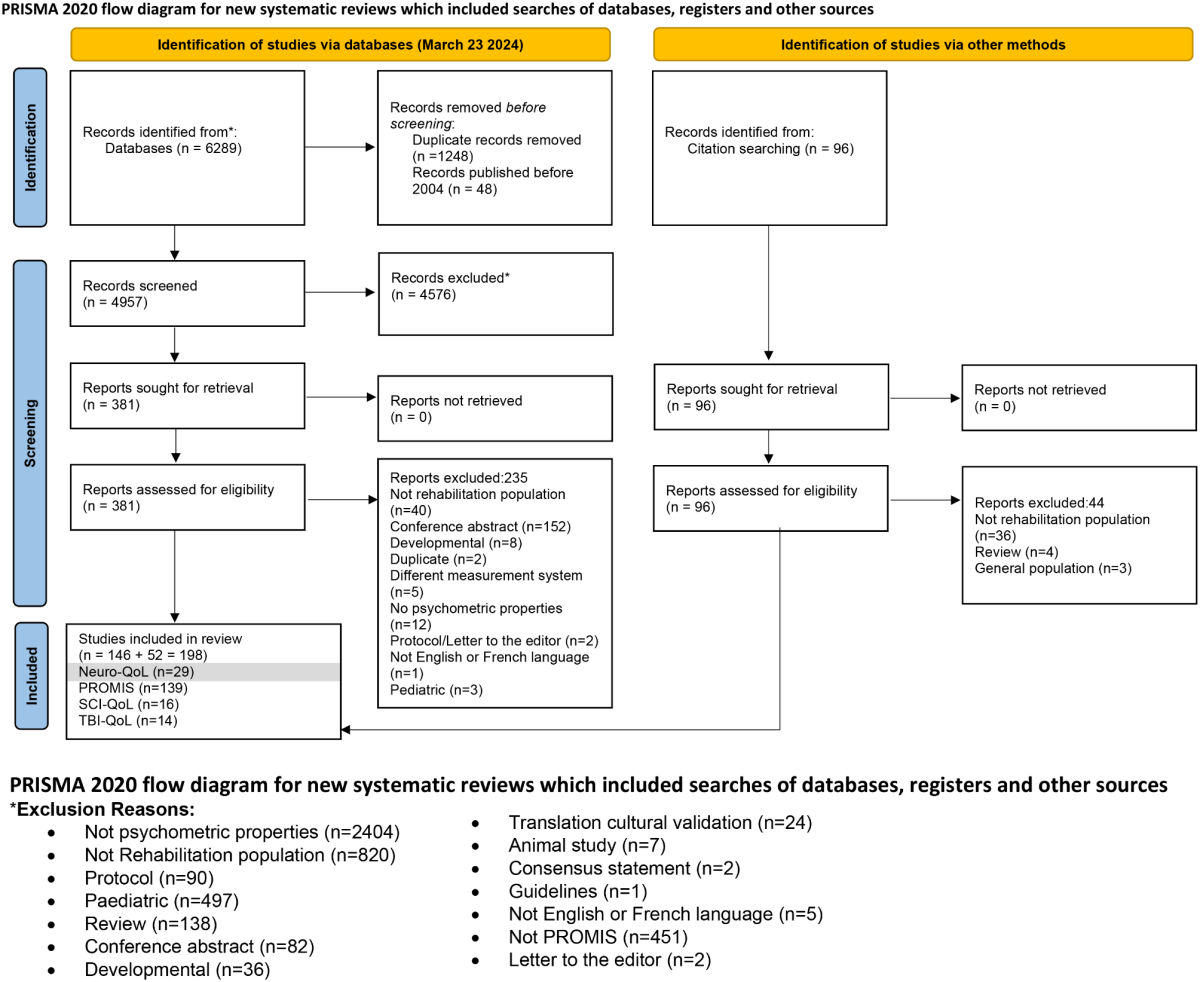
PRISMA 2020 flow diagram

We retrieved a total of 6289 articles and 4957 articles remained following deduplication. Title and abstract screening resulted in 381 included articles. The full-text screen resulted in 146 included articles and reference checks resulted in an additional 52 included articles for a total of 198 included articles. Twenty-nine of these were Neuro-QoL articles and of these, three were identified in the reference checks (Fig. 1).

Authors from all but two studies [26, 27] collected data from the United States only. In alphabetical order, patient populations included amyotrophic lateral sclerosis (n = 3), cognitive decline or mild cognitive impairment (n = 2), Huntington’s disease (n = 8), lupus (n = 1), mixed neurological conditions (n = 3), multiple sclerosis (n = 8), Parkinson’s disease (n = 4) and stroke (n = 8). Some studies included a population with mixed diagnoses. The full study and population characteristics can be found within the extraction table (Appendix C). We divided the results in each Neuro-QoL domain according to diagnosis (Table 2, Appendix D).

**Table 2:** Results of COSMIN synthesis

		COSMIN									IRT or Rasch			
Domain	Type	# articles	Structural validity	Internal consistency	Reliability	Measurement error	Cross- cultural validity	Criterion validity	Construct validity	Responsiveness	Structural validity	Person, item reliability, separation	Cross- cultural validity	Wright map
*Amyotrophic lateral sclerosis (ALS)*														
Fatigue	SF	1							+; H					
*Cognitive decline or mild cognitive impairment*														
Cognitive function	SF	1							?; M	+; M				
Cognitive function *Executive function (v1.0)	SF	1			-; L				+; H	+; H				
*Huntington’s disease*														
Ability to participate in social roles and activities	SF	2		?; H	+; H				+; H	+; H				
Ability to participate in social roles and activities	CAT	2			+; H				+; H	+; H				
Anxiety	CAT	1			+; H	-; H				+; H				
Cognitive function	SF	1			+; H					+; M				
Cognitive function	CAT	1			+; H					+; M				
Cognitive function, executive function (v1.0)	SF	1		?; H				+; H	+; H					
Cognitive function, general cognitive concerns (v1.0)	SF	1		?; H				-; H	-; H					
Depression	CAT	1			+; M	-; M				+; M				
Emotional and behavioural dyscontrol	SF	1			+; M	-; M				+; M				
Emotional and behavioural dyscontrol	CAT	1			+; M	+; M				+; M				
Lower extremity function—mobility	SF	2		?; H	+; M	+; M				+; M				
Lower extremity function—mobility	CAT	2			+; M	+; H		+; H	-; H	+; M				
Positive affect and well-being	SF	1			+; M	-; M				+; M				
Positive affect and well-being	CAT	1			+; M	-; M				+; M				
Satisfaction with social roles and activities	SF	2			+; M				+; H	+; M				
Satisfaction with social roles and activities	CAT	2			+; M					+; M				
Stigma	SF	1			+; M	-; M				+; M				
Stigma	CAT	1			+; M	+; H				+; M				
Upper extremity	SF	2		?; H	+; M	-; M				?; M				
Upper extremity	CAT	2			+; M	+; H		+; H	+; H	+; M				
*Lupus*														
Cognitive function*executive function (v1.0)	SF	1			+; H									
Cognitive function*general cognitive concerns (v1.0)	SF	1			+; H									
*Mixed neurological conditions*														
Ability to participate in social roles and activities	SF	1		?; H					+; H	+; H				
Cognitive function	SF	1		?; H					+; H					
Cognitive function, executive function (v1.0)	SF	1		?; H					+; H	+; H				
Depression	SF	1		?; H					+; H	+; H				
Lower extremity function—mobility	SF	1		?; H					+; H	+; H				
*Multiple sclerosis*														
Total scale	profile	1							+; H					
Ability to participate in social roles and activities	SF	2	-; H	-; H	+; H				+; H					
Anxiety	SF	2	-; H	-; H	+; H				+; H					
Cognitive function, executive function (v1.0)	SF	2	-; H	-; H	+; H				+; H					
Cognitive function, general cognitive concerns (v1.0)	SF	2	+; H	+; H	+; H				+; H					
Communication	scale	1	+; H	+; H					+; H					
Depression	SF	2	+; H	+; H	+; H				+; H					
Emotional and Behavioural dyscontrol	SF	2	-; H	-; H	+; H				+; H					
Fatigue	SF	2	-; H	-; H	+; H				+; H					
Lower extremity function—mobility	SF	2	-; H	-; H	+; H				+; H					
Positive affect and well-being	SF	2	-; H	-; H	+; H				+; H					
Satisfaction with social roles and activities	SF	1	+; H		+; H				+; H					
Sleep disturbance	SF	2	-; H	-; H	+; H				+; H					
Stigma	SF	1		-; H	+; H				+; H					
Upper extremity	SF	2	-; H	-; H	+; H				+; H					
*Parkinson’s disease*														
Anxiety	SF	1		-; H	+; M	+; M			?; H	+; H				
Depression	SF	1		-; H	+; M	+; M			?; H	+; H				
Cognitive Function, executive function (v1.0)	SF	1		-; H	+; M	+; M			+; H	+; H				
Cognitive function, general cognitive concerns (v1.0)	SF	1		-; H	+; M	+; M			+; H	+; H				
Emotional and behavioural dyscontrol	SF	1		-; H	+; M	+; M			?; H	+; H				
Fatigue	SF	1		-; H	+; M	+; M			+; H	+; H				
Lower extremity function—mobility	SF	1		-; H	+; M	+; M			+; H	+; H				
Positive affect and well-being	SF	1		-; H	+; M	+; M			+; H	+; H				
Satisfaction with social roles and activities	SF	1		-; H	+; M	+; M			+; H	+; H				
Sleep disturbance	SF	1		-; H	+; M	+; M			+; H	+; H				
Stigma	SF	1		-; H	+; M	+; M			+; H	+; H				
Upper extremity	SF	1		-; H	+; M	+; M			+; H	+; H				
*Stroke*														
Ability to participate in social roles and activities	SF	1			-; H									
Ability to participate in social roles and activities	Item bank	1									?; M			
Anxiety	SF	1			-; H									
Cognitive function	SF	3	-; H	-; H	-; H			-; H	+; H					
Cognitive function, executive function (v1.0)	SF	1			-; H									
Cognitive function, general cognitive concerns (v1.0)	SF	1			-; H									
Depression	SF	1			-; H									
Emotional and behavioural dyscontrol	SF	1			’-; H									
Fatigue	SF	1			’-; H									
Lower extremity function—mobility	SF	1			’-; H									
Positive affect and well-being	SF	1			’-; H									
Positive psychological function	Item bank	1							+; H		?; M	Person, item rel. > 0.8		
Satisfaction with social roles and activities	SF	2			’-; H									
Satisfaction with social roles and activities	Item bank	1									?; M			
Sleep disturbance	SF	2	-; H	-; H	’-; H									
Stigma	SF	1			’-; H									
Upper extremity	SF	1			’-; H									

*CAT* computerized adaptive test, *SF* short form

GRADE approach: *H* high, *M* moderate, *L* low; *VL* very low

Measurement property rating: + Sufficient; − Insufficient;? Indeterminant

*Person Item Rel.* person item reliability, *IRT* item response theory

*All domains are the current version (v2.0), which have not changed from the original version (v1.0). The exceptions are the Cognitive Function, General Cognitive Concerns (v1.0) and Cognitive Function, Executive Function domains (v1.0). These were combined into Cognitive Function (v2.0) in the version 2 update

### Content Validity of Neuro-QoL

The original development of Neuro-QoL included Alzheimer’s disease, multiple sclerosis, amyotrophic lateral sclerosis, Parkinson’s disease, stroke, and adult and pediatric epilepsy conditions, and has been described extensively [5, 28–32]. The results from these studies indicate that Neuro-QoL domains possess sufficient content validity, encompassing comprehensibility, relevance, and comprehensiveness, based on high-quality evidence across these diagnoses. Since the initial Neuro-QoL development, only one article added to the evidence for the content validity of Neuro-QoL in rehabilitation. The results of this article indicate sufficient comprehensibility of the Spanish version of all Neuro-QoL domains across neurological conditions based on high-quality evidence from cognitive debriefing with patients and caregivers [27].

### Amyotrophic lateral sclerosis

Evidence from the single article reporting measurement properties for amyotrophic lateral sclerosis indicates that the Neuro-QoL fatigue domain has sufficient construct validity (2 out of 2 tested hypotheses met) based on high-quality evidence [33].

### Cognitive decline or mild cognitive impairment

In individuals at risk for cognitive decline, construct validity for the cognitive function domain (v2.0) is currently indeterminant (2/3 hypotheses met) and responsiveness sufficient (4/5 hypotheses met) based on moderate quality evidence due to a low sample size (n = 76) [34]. The other evidence available for this subgroup is for the executive function domain (v1.0), which was combined with general concerns (v1.0) to form the cognitive function domain in version 2.0. For individuals already assessed with mild cognitive impairment, there is sufficient evidence for construct validity (1/1 hypotheses met) and responsiveness (1/1 hypotheses met) based on high-quality evidence. However, test-retest reliability is currently assessed as insufficient (r = 0.35) based on low-quality evidence since the authors provided inadequate information on patient stability between tests and they calculated a correlation coefficient as opposed to an intraclass correlation coefficient [35].

### Huntington’s disease

For the ability to participate in social roles and activities domain, both the short form and CAT have sufficient evidence for test-retest reliability, construct validity (4/4 and 8/11 hypotheses met, respectively), and responsiveness (5/6 and 6/6 hypotheses met, respectively) [36, 37]. Currently, the internal consistency of the short form receives an indeterminate rating due to the lack of structural validity evidence, despite having a Cronbach’s alpha greater than 0.7 (specifically 0.94) [37].

For the anxiety CAT, the emotional and behavioral dyscontrol short form and CAT and positive affect and wellbeing short form and CAT, there is sufficient evidence for test-retest reliability and responsiveness (all 2/2 hypotheses met) based on moderate-high quality evidence (low sample sizes). While the emotional and behavioural dyscontrol CAT has evidence of sufficient measurement error, measurement error for the emotional and behavioural dyscontrol short form, anxiety CAT and positive affect and wellbeing short form and CAT is rated as insufficient because the standard error of measurement (SEM) (3.11, 1.83, 1.72, 2.41 respectively) is greater than the minimal important change (MIC) (2.65 ± 8.22, 0.62 ± 6.08, −0.91 ± 5.28, −1.25 ± 6.67) [38].

For the cognitive function (v2.0) domain, both the short form and CAT demonstrate sufficient test-retest reliability and responsiveness (1/1 hypothesis met) [39]. The executive function v1.0 short form demonstrates sufficient criterion and construct validity (3/3 hypotheses met) while the general cognitive concerns short form currently has insufficient (AUC = 0.68) and indeterminant evidence (2/3 hypotheses met), respectively [40]. Both of these v1.0 short forms currently lack structural validity evidence, meaning that internal consistency currently receives an indeterminant rating despite both forms having a Cronbach’s Alpha greater than 0.7 [40].

The depression CAT has evidence for sufficient test-retest reliability and construct validity (2/2 hypotheses met) based on moderate quality evidence. However, since the SEM (1.85) is greater than the MIC (1.68 ± 8.90), measurement error is currently rated as insufficient based on moderate quality evidence [38].

The lower extremity function—mobility short form and CAT have sufficient evidence of test-retest reliability, measurement error, and responsiveness (9/12 and 10/12 hypotheses met, respectively) based on moderate or high-quality evidence [41, 42]. Currently, the short form is assigned an indeterminate rating for internal consistency due to the absence of evidence for structural validity, even though it has a Cronbach’s Alpha greater than 0.7, specifically 0.93 [40]. The CAT has sufficient evidence for criterion validity (3/3 equations with the area under the curve (AUC) greater than 0.7) but indeterminant construct validity (8/11 hypotheses met) based on high-quality evidence [41].

The satisfaction with social roles and activities and stigma short forms and CATs have sufficient evidence of test-retest reliability and responsiveness (2/2, 9/11 hypotheses met and 2/2, 9/11 hypotheses met, respectively) based on moderate quality evidence due to small sample sizes [36–38]. The satisfaction with social roles and activities short form also has sufficient construct validity based on high-quality evidence (4/4 hypotheses met) [37]. While the stigma CAT has sufficient measurement error, the SEM for the short form is insufficient (SEM = 2.54, MIC = 1.43 ± 4.62) [38].

For the upper extremity domain, both the short form and CAT have sufficient evidence of test-retest reliability based on moderate quality evidence due to small sample sizes [41, 42]. The CAT also has sufficient evidence for measurement error, criterion validity (3/3 AUC > 0.7) and construct validity (9/11) based on high-quality evidence. In contrast, the short form currently has insufficient evidence for measurement error (SEM: 3.82, MIC decline: −2.87 (6.89), MIC improvement: −0.25 (7.16)). Furthermore, there is not yet consistent evidence to make a definitive rating for internal consistency (Cronbach’s alpha 0.94 but no structural validity evidence) or responsiveness (8/11 hypotheses met).

### Lupus

In the executive function and general cognitive concerns (v1.0) domains, the short form has sufficient evidence for test-retest reliability [43].

### Mixed neurological conditions

For a mixed neurological group including individuals with epilepsy, stroke, amyotrophic lateral sclerosis, multiple sclerosis, and Parkinson’s disease, the short forms for the ability to participate in social roles and activities, executive function (v1.0), depression, and lower extremity domains have evidence for sufficient construct validity (1/1 hypothesis met) and responsiveness (1/1 hypothesis met) [44]. For the cognitive function (v2.0) domain, there is sufficient evidence for construct validity only (1/1 hypothesis met) [45]. For all of these domains, internal consistency is indeterminant due to a current lack of evidence for structural validity [44, 45].

### Multiple sclerosis

The total Neuro-QoL scale (i.e., all domains together) has sufficient construct validity based on high-quality evidence (1/1 hypothesis met) [26].

The ability to participate in social roles and activities and anxiety short forms have sufficient evidence of test-retest reliability and construct validity [46, 47]. However, despite Cronbach’s alpha being greater than 0.7 (0.89 [46], 0.96 [47] and 0.93 [46], 0.94 [47], respectively), evidence for insufficient structural validity due to both the comparative fit index and Tucker-Lewis index being less than 0.95 and the root mean square error of approximation greater than 0.06 [47] currently results in an insufficient rating for internal consistency.

The two cognitive function short forms from v1.0 of Neuro-QoL (i.e. executive function and general cognitive concerns) have evidence for sufficient test-retest reliability [46] and construct validity based on high-quality evidence (15/17 hypotheses met) [46, 47]. However, executive function currently has evidence of insufficient structural validity (CFI and TFI < 0.95) [47], thus resulting in an insufficient rating for internal consistency as well [46, 47].

For the communication scale and depression short form, there is sufficient evidence for structural validity [47], internal consistency [46, 47] and construct validity (15/17 hypotheses met) [46, 47]. The depression short form also has sufficient evidence of test-retest reliability based on high-quality evidence [46].

The fatigue short form has sufficient evidence of test-retest reliability and construct validity (13/17 hypothesis met) [47]. However, despite Cronbach’s alpha being greater that 0.7 (0.93 [47]), evidence for insufficient structural validity due to both CFI and TLI being less than 0.95 [47] currently results in an insufficient rating for internal consistency too. Measurement error for the fatigue short form is currently indeterminant because the MIC has not been calculated [48].

The emotional and behavioral dyscontrol, fatigue, lower extremity—mobility, positive affect and well-being, sleep disturbance, stigma, and upper extremity domains all have sufficient evidence for test-retest reliability and construct validity (13–16/17 hypotheses met). Current evidence of insufficient structural validity (CFI and TLI < 0.95) or a lack of evidence (stigma) currently results in insufficient evidence for internal consistency despite Cronbach’s alpha being > 0.7 in all domains [46, 47].

### Parkinson’s disease

The anxiety, depression, cognitive function v1.0 forms (executive function and general cognitive concerns), emotional and behavioural dyscontrol, fatigue, lower extremity—mobility, positive affect and wellbeing, satisfaction with social roles and activities, sleep disturbance, stigma, and upper extremity short forms have sufficient evidence for test-retest reliability, measurement error and responsiveness (1/1 hypotheses met) based on moderate to high-quality evidence. For all domains, Cronbach’s alpha is > 0.7 but without evidence of structural validity, internal consistency is currently rated as insufficient. All domains but anxiety, depression, and emotional and behavioral dyscontrol have sufficient evidence for construct validity (ranging from 8–10/11 hypotheses met). These three domains currently have indeterminant evidence for construct validity (6–7/11 hypotheses met) [49].

### Stroke

The ability to participate in social roles and activities short form has insufficient evidence of inter-rater reliability between patients and proxy raters (ICC = 0.55) based on high-quality evidence [50]. The ability to participate in social roles and activities item bank has indeterminant structural validity based on moderate quality evidence because information on monotonicity was not reported [51].

The cognitive function (v2.0) [52–54] domain has sufficient evidence for construct validity (3/3 hypotheses met). There is not yet enough evidence for sufficient structural validity (RMSEA > 0.06) [52], inter-rater reliability between patients and proxies (ICC = 0.54) [53] internal consistency (Cronbach’s alpha > 0.7 but insufficient evidence for structural validity) [52] or criterion validity (AUC = 0.691) [50]. The executive function (v1.0) and general cognitive concerns (v1.0) domains currently have insufficient evidence for inter-rater reliability between patients and proxies (ICC = 0.56, 0.59, respectively) [50].

The short forms for anxiety, depression, emotional and behavioural dyscontrol, fatigue, mobility, positive affect and wellbeing, satisfaction with social roles and activities, sleep disturbance, stigma and upper extremity have evidence for insufficient inter-rater reliability between patients and proxies (ICC = 0.32–0.53) [50]. The sleep disturbance short form has insufficient structural validity due to RMSEA not meeting cutoffs (RMSEA = 0.12). Due to insufficient structural validity, internal consistency is also insufficient [52].

The positive psychological function and satisfaction with social roles and activities item banks have indeterminant evidence of structural validity because monotonicity was not reported. Positive psychological function person and item reliability meet Rasch criteria (> 0.8) [51].

## Discussion

We performed a systematic review to assess the strength and quality of the measurement properties of Neuro-QoL. We reported the measurement properties of Neuro-QoL according to diagnosis (amyotrophic lateral sclerosis, cognitive decline or mild impairment, Huntington’s disease, lupus, mixed neurological conditions, multiple sclerosis, Parkinson’s disease and stroke) and domain (e.g., fatigue, stigma, etc.). Consequently, this manuscript serves as a comprehensive reference for researchers and clinicians, offering diagnosis-specific recommendations for the application of Neuro-QoL. All results and associated recommendations are based on COSMIN’s rating system according to currently available evidence. COSMIN’s requirements are more extensive and stringent than others in the field (e.g., International Society for Quality of Life Research (ISOQOL) [55], International Society for Pharmacoeconomics and Outcomes Research (ISPOR) [56, 57], HealthMeasures reporting standards [58]). For example, ISOQOL provides guidance concerning the minimal measurement properties and associated statistical tests recommended for the use of patient-reported outcome measures in patient-centered outcome research [55]. COSMIN does the same but goes a step further by providing cut-off scores to rate evidence sufficiency and criteria to assess evidence quality. The ratings and recommendations we made in this review based on COSMIN provide guidance regarding gaps in the available evidence. As such, the recommendations in this review are subject to change as new evidence becomes available.

In most cases, there is only one article providing measurement property evidence for each domain in each diagnosis. However, this limited evidence is strong as most studies’ measurement properties were rated sufficient based on high-quality evidence. As a result, our general recommendation is that Neuro-QoL is appropriate for use across various neurological conditions, per HealthMeasures guidelines [59, 60]. Specific recommendations for each diagnosis are detailed in Table 3.

**Table 3:** Specific recommendations for the use of Neuro-QoL according to the results of this review

Diagnoses	Current evidence indicates can be used to	More evidence required to
*Amyotrophic lateral sclerosis (ALS)*	Appropriately gather information from this population Describe patient fatigue at admission	Evaluate or predict patient fatigue Describe, evaluate or predict patient outcomes on all other relevant domains
*Cognitive decline or mild cognitive impairment*	Appropriately gather information from this population Describe and evaluate a patient’s change in cognitive function over time	Evaluate or predict patient cognitive function Describe, evaluate or predict patient outcomes on all other relevant domains
*Huntington’s disease*	Appropriately gather information from this population Describe and evaluate a patient’s change over time in 10 domains (ability to participate in social roles and activities, anxiety, cognitive function, depression, emotional and behavioral dyscontrol, lower extremity function—mobility, positive affect and wellbeing, satisfaction with social roles and activities, stigma, upper extremity) Distinguish between individuals with cognitive impairment, lower extremity—mobility impairment and upper extremity impairment versus those without	To predict patient outcomes on all domains
*Lupus*	Appropriately gather information from this population	Describe, evaluate or predict patient outcomes on all relevant domains
*Mixed neurological conditions*	Appropriately gather information from this population Describe a patient’s cognitive function Describe and evaluate a patient’s change over time in 4 domains (ability to participate in social roles and activities, cognitive function—executive function, depression, lower extremity function—mobility)	Describe, evaluate or predict patient outcomes on all other relevant domains
*Multiple sclerosis*	Appropriately gather information from this population Describe and evaluate a patient at a single timepoint in 12 domains (ability to participate in social roles and activities, anxiety, cognitive function, communication, depression, emotional and behavioral dyscontrol, fatigue, lower extremity function—mobility, positive affect and wellbeing, sleep disturbance, stigma, upper extremity)	To predict patient outcomes on all domains
*Parkinson’s disease*	Appropriately gather information from this population Describe and evaluate a patient over time in 11 domains (anxiety, cognitive function, depression, emotional and behavioral dyscontrol, fatigue, lower extremity function—mobility, positive affect and wellbeing, satisfaction with social roles and activities, sleep disturbance, stigma, upper extremity)	To predict patient outcomes on all domains
*Stroke*	Appropriately gather information from this population Describe a patient’s cognitive function and positive psychological function at a single timepoint	Evaluate and predict a patient’s cognitive function and positive psychological function Describe, evaluate or predict patient outcomes on all other relevant domains

The least amount of evidence is available for the diagnoses of amyotrophic lateral sclerosis, cognitive decline or mild impairment, lupus, and mixed neurological conditions, both in terms of the evidence for different measurement properties and domains. For example, despite amyotrophic lateral sclerosis being a core neurological condition for which Neuro-QoL was developed, the only measurement property evidence available in rehabilitation, aside from content validity, pertains to the construct validity of the fatigue short form [33]. Rehabilitation researchers and clinicians are currently limited in the domains and interpretations they can make based on research evidence when using Neuro-QoL within these populations.

In contrast, Huntington’s disease, multiple sclerosis, Parkinson’s disease and to a lesser extent, stroke have a range of measurement evidence available across most (though not all,) Neuro-QoL domains. Of these, only Huntington’s disease has evidence for CATs [37, 38]. Although evidence for these diagnoses is typically confined to 1–2 articles per domain, these articles often offer high-quality evidence for different measurement properties. Thus, rehabilitation researchers and clinicians have more information available to justify their use and interpretation of Neuro-QoL in these domains. However, a notable gap is the lack of predictive use, such as employing admission scores to forecast discharge or follow-up outcomes. Further research in this area may be particularly fruitful.

There were some patterns in measurement properties ratings across domains or diagnosis within this review which suggest avenues for future research. For example, criterion and construct validity and responsiveness tend to be sufficient in Huntington’s disease, multiple sclerosis, Parkinson’s disease and stroke. Additionally, across various conditions, it’s common to find that Cronbach’s alpha exceeds 0.7. However, it is often rated as insufficient, typically due to either a lack of adequate structural validity evidence or the presence of evidence indicating insufficient structural validity [46, 47]. The only instances where there is sufficient structural validity evidence was for general cognitive concerns and depression short forms, and the communication scale when used with individuals with multiple sclerosis [47]. The structural validity of Neuro-QoL domains in rehabilitation populations is a key area for future research.

There is either test-retest (Huntington’s disease, multiple sclerosis, Parkinson’s disease) or inter-rater (stroke) reliability evidence available. In all populations test-retest reliability was sufficient (ICC > 0.7) and inter-rater reliability between patients and proxies was insufficient (ICC < 0.7). Inter-rater reliability for all 12 short forms used in stroke may be negatively affected due to stroke’s effects on patients’ self-awareness, and vision, hearing, and cognitive status, all of which may contribute to patients responding differently from caregivers’ [61]. Future research should consider these patient variables so that Neuro-QoL can be interpreted more holistically.

Measurement error evidence was available for Neuro-QoL domains when used in Huntington’s disease and Parkinson’s disease. While Parkinson’s disease has evidence for sufficient construct validity across all domains [49], the evidence is mixed for Huntington’s disease because the SEM is often greater than the MIC [38]. Further estimates of both SEM and MIC may clarify this measurement property, allowing researchers and clinicians to interpret change scores more confidently.

There are some Neuro-QoL domains (e.g., bowel function, sexual function, urinary/bladder function [7, 8]) that have no evidence across any of the rehabilitation populations studied. In this case, these are only item pools and no Neuro-QoL measure has yet been developed for these domains. Similarly, there are some instances where an article was included but the evidence was uninterpretable, resulting in no evidence for that diagnosis and domain. For example, an article containing evidence of the construct validity of the Neuro-QoL CAT for stroke patients only reported p values rather than correlations or other statistics providing construct validity evidence [62]. Researchers should consider generating evidence for these Neuro-QoL diagnoses and domains for which there is currently no evidence available in rehabilitation.

This Neuro-QoL review is mirrored by complementary systematic reviews that we completed of the [1] interpretability of Neuro-QoL, PROMIS, TBI-QoL and SCI-QoL [2], measurement properties of PROMIS and [3] measurement properties of TBI-QoL and SCI-QoL. These reviews demonstrate that there is some overlap between the use of Neuro-QoL and PROMIS. Specifically, PROMIS has been used by patients with a stroke, multiple sclerosis, Parkinson’s disease, Huntington’s disease and amyotrophic lateral sclerosis diagnoses. When the same PROMIS and Neuro-QoL domains have been used for one of these patient groups the results are similar, indicating that the overlapping PROMIS and Neuro-QoL domains could potentially be used for these populations. Of note is that there are psychometric properties reported for PROMIS anxiety, depression, fatigue and physical function CATs when used by individuals with multiple sclerosis [63], while there is no psychometric evidence for Neuro-QoL domain CATs for this group. There is also far more data available concerning the psychometric properties of PROMIS domains rather than Neuro-QoL domains for individuals with lupus—and the available data indicates the measurement properties of the PROMIS domains are sufficient based on high-quality evidence. This may be because, while lupus has neurological effects, it is not a neurological condition but an autoimmune disorder. Consequently, most Neuro-QoL domains may not be appropriate for individual with lupus. The opposite is true for Huntington’s disease with a much greater amount of high-quality evidence available for Neuro-QoL domains rather than PROMIS. For both Neuro-QoL and PROMIS domains, there is little psychometric information available for their use by patients with ALS.

Finally, there were two recurring methodological concerns across studies included in this review which often reduced the methodological quality of evidence for certain measurement properties per COSMIN criteria. First, the authors did not provide hypotheses for construct validity or responsiveness with both magnitude and direction. Per COSMIN guidelines, the authors of this review had to assign testable hypotheses for the evidence from these studies to be interpretable [23]. We recommend that researchers investigating construct validity between Neuro-QoL and other measures use the a priori hypotheses that we developed for this review (Appendix B). We also noted issues with reporting adequate details on the stability of patients between administrations when estimating test-retest reliability and reporting all statistics concerning model fit for Rasch and IRT models (i.e., to support monotonicity, local independence, fit and unidimensionality [16]). Ensuring this information is reported in future manuscripts concerning the measurement properties of Neuro-QoL domains would advance our understanding of this measurement system.

## Strengths and limitations

The subgroups for synthesis in this systematic review were developed with the aim to provide information that is as relevant and specific as possible. However, the evidence base isn’t large for each of these subgroups—ranging from 1–3 articles in each. Furthermore, as mentioned earlier, recurring methodological concerns reduced the quality of these studies. While we were able to address some of these concerns, such as setting a priori hypotheses, there remains a need for high-quality research to replicate and expand upon the existing evidence.

Although COSMIN’s systematic review guidance is extensive, we had to extend COSMIN’s guidance to encompass the Rasch analyses used by some Neuro-QoL studies. Our extensions represent deviations from COSMIN’s protocol; however, they can also inform systematic review authors in their future work [24, 64]. Future work linking Rasch estimates to classical test theory statistics within COSMIN would be an important methodological development.

## Conclusion

The evidence suggests that rehabilitation researchers and clinicians can use most Neuro-QoL domains in Huntington’s disease, multiple sclerosis, Parkinson’s disease and stroke to describe and evaluate patients. There is evidence in a limited number of Neuro-QoL domains for its use as a descriptive measure only in amyotrophic lateral sclerosis, cognitive decline or mild impairment, lupus, and mixed neurological conditions. Since all diagnoses and domains only have 1–3 articles providing evidence, further investigation of Neuro-QoL measurement properties across neurological conditions in rehabilitation would be beneficial. Evidence of structural validity, measurement error, cross-cultural validity, and predictive validity would advance the use and interpretation of Neuro-QoL in rehabilitation.

## Electronic supplementary material

Below is the link to the electronic supplementary material.

Supplementary Material 1

Supplementary Material 2

Supplementary Material 3

Supplementary Material 4

## Data Availability

All data is found in the appendices, as extracted from published research articles.

## Abbreviations

Neuro-QoL: Quality of Life in Neurological Disorders measurement system
CAT: Computerized adaptive testing
PROMIS: Patient reported outcomes measurement information system
TBI-QoL: Traumatic brain injury quality of life
SCI-QoL: Spinal cord injury quality of life
COSMIN: COnsensus-based standards for the selection of health Measurement Instruments
GRADE: Grading of recommendations assessment, development and evaluation
SEM: Standard error of measurement
MIC: Minimal important change
ISOQOL: International society for quality of life research
ISPOR: International society for pharmacoeconomics and outcomes research
MEDLINE: Medical literature analysis and retrieval system online
HaPi: Health and psychological instruments
CINAHL: Cumulative index to nursing and allied health literature
EBSCO: Elton B. Stephens company research database
ISRCTN: International standard randomised controlled trial number
EMBASE: Excerpta medica database
