# Supplementary material for: The psychometric properties of the Quality of Life in Neurological Disorders (Neuro-QoL) measurement system in neurorehabilitation populations: a systematic review
Source: J Patient Rep Outcomes. 2024 Sep 18;8:106. doi: 10.1186/s41687-024-00743-7 (PMC11410750; doi:10.1186/s41687-024-00743-7)
Supplement: Supplementary file 1 — Supplementary Material 1 [file 41687_2024_743_MOESM1_ESM.doc]

Database: Ovid MEDLINE(R) ALL <1946 to November 15, 2022>

Search Strategy:

--------------------------------------------------------------------------------

1 (instrumentation or methods).fs. or (Validation Studies or Comparative Study).pt. or exp Psychometrics/ or psychometr*.ti,ab. or (clinimetr* or clinometr*).tw. or exp Outcome Assessment, Health Care/ or outcome assessment.ti,ab. or outcome measure*.tw. or exp Observer Variation/ or observer variation.ti,ab. or exp Health Status Indicators/ or exp Reproducibility of Results/ or reproducib*.ti,ab. or exp Discriminant Analysis/ or (reliab* or unreliab* or valid* or coefficient or homogeneity or homogeneous or internal consistency).ti,ab. or (cronbach* and (alpha or alphas)).ti,ab. or (item and (correlation* or selection* or reduction*)).ti,ab. or (agreement or precision or imprecision or precise values or test-retest).ti,ab. or (test and retest).ti,ab. or (reliab* and (test or retest)).ti,ab. or (((replicab* or repeated) and (measure or measures or findings or result or results or test or tests)) or (generaliza* or generalisa* or concordance) or (intraclass and correlation*) or (discriminative or known group or factor analysis or factor analyses or dimension* or subscale*) or (multitrait and scaling and (analysis or analyses)) or (item discriminant or interscale correlation* or error or errors or individual variability) or (variability and (analysis or values)) or (uncertainty and (measurement or measuring)) or (standard error of measurement or sensitiv* or responsive*) or ((minimal or minimally or clinical or clinically) and (important or significant or detectable) and (change or difference)) or (small* and (real or detectable) and (change or difference)) or (meaningful change or ceiling effect or floor effect or Item response model or IRT or Rasch or Differential item functioning or DIF or computer adaptive testing or item bank or cross-cultural equivalence)).ti,ab. or (stability or interrater or inter-rater or intrarater or intra-rater or intertester or inter-tester or intratester or intra-tester or interobserver or inter-observer or intraobserver or intraobserver or intertechnician or inter-technician or intratechnician or intra-technician or interexaminer or inter-examiner or intraexaminer or intra-examiner or interassay or interassay or intraassay or intra-assay or interindividual or inter-individual or intraindividual or intra-individual or interparticipant or inter-participant or intraparticipant or intra-participant or kappa or kappas or kappas or repeatab*).ti,ab. (10378421)

2 Geriatric*.mp. or exp Geriatrics/ or exp Cardiovascular Diseases/ or cardiorespiratory.mp. or neurological.mp. or nervous system disease.mp. or exp Nervous System Diseases/ or orthopedic.mp. or exp Orthopedics/ or chronic condition*.mp. or exp chronic condition/ (5027419)

3 (PROMIS or patient reported outcome measurement information system or Quality of Life in Neurologic Disorders or Neuro QoL or Traumatic Brain Injury Quality of Life or TBI QoL or Spinal Cord Injury Quality of Life or Sci QoL).mp. (2805)

4 and/1-3 (619)
